# Innovative microwave in situ approach for crystallizing TiO_2_ nanoparticles with enhanced activity in photocatalytic and photovoltaic applications

**DOI:** 10.1038/s41598-024-63614-7

**Published:** 2024-06-01

**Authors:** Adam Kubiak, Maciej Zalas, Michał Cegłowski

**Affiliations:** Faculty of Chemistry, Adam Mickiewicz University, Poznan, Uniwersytetu Poznanskiego 8, 61614 Poznan, Poland

**Keywords:** Titanium dioxide, Photocatalysis, Formic acid, Photovoltaic, DSSC, Multifunctional application, Materials chemistry, Photocatalysis

## Abstract

This investigation introduces an innovative approach to microwave-assisted crystallization of titania nanoparticles, leveraging an in situ process to expedite anatase crystallization during microwave treatment. Notably, this technique enables the attainment of crystalline material at temperatures below 100 °C. The physicochemical properties, including crystallinity, morphology, and textural properties, of the synthesized TiO_2_ nanomaterials show a clear dependence on the microwave crystallization temperature. The presented microwave crystallization methodology is environmentally sustainable, owing to heightened energy efficiency and remarkably brief processing durations. The synthesized TiO_2_ nanoparticles exhibit significant effectiveness in removing formic acid, confirming their practical utility. The highest efficiency of formic acid photodegradation was demonstrated by the T_200 material, reaching almost 100% efficiency after 30 min of irradiation. Furthermore, these materials find impactful application in dye-sensitized solar cells, illustrating a secondary avenue for the utilization of the synthesized nanomaterials. Photovoltaic characterization of assembled DSSC devices reveals that the T_100 material, synthesized at a higher temperature, exhibits the highest photoconversion efficiency attributed to its outstanding photocurrent density. This study underscores the critical importance of environmental sustainability in the realm of materials science, highlighting that through judicious management of the synthesis method, it becomes feasible to advance towards the creation of multifunctional materials.

## Introduction

In the face of escalating environmental concerns and the imperative to embrace sustainable practices, pursuing innovative technologies becomes increasingly critical^1,2^. This publication explores a novel avenue of material synthesis, focusing on the crystallization of TiO_2_ nanoparticles through the pioneering approach of microwave in situ synthesis.

The urgency to transition towards green chemistry is underscored by the adverse effects of escalating pollutant emissions on our environment and the pressing need for clean energy solutions^3–5^. While significant strides are being made in altering top-down approaches to energy production, a complementary bottom-up approach becomes indispensable. Yang et al.^6^ emphasize the critical need to explore groundbreaking technologies within materials science, particularly nanomaterials exhibiting precisely defined morphology, crystallinity, and textural properties^7,8^. These materials hold the key to addressing the challenges posed by sustainable development and zero-emission strategies across scientific and industrial domains^9,10^.

Titanium dioxide emerges as a crucial material in the domain of photocatalysis, owing to its exceptional properties such as robust oxidizing abilities, superhydrophilicity, and chemical stability^11,12^. The photocatalytic activity of TiO_2_ hinges on generating charge carriers upon UV light absorption, facilitating the degradation of organic pollutants. However, optimizing efficiency necessitates meticulous control over particle size, surface area, pore structure, and crystalline phase^13,14^. Therefore, developing performance improvements by adjusting these factors remains the focus of photocatalysis research.

In materials science, the microwave-assisted hydrothermal method has emerged as a pioneering approach for synthesizing TiO_2_ nanoparticles, presenting a transformative pathway for enhancing their efficiency in photocatalytic applications^15,16^. Integrating microwaves into the hydrothermal reactor accelerates heating and overcomes the limitations of slow convection and heat conduction associated with traditional methods. Microwave synthesis reduces energy costs and enables rapid reaction mechanisms, resulting in high-yield, pure products in significantly shorter timescales. Dar et al.^17^ reported that a microwave-based approach achieves ~ 7 nm and ∼100–400 nm nanostructures of anatase titania. On the other hand, an important conclusion was presented by Kubiak et al.^18^, who obtained TiO_2_ in the microwave process supported by surfactants. The research team confirmed that the obtained materials were characterized by a reduction in the bandgap energy concerning the literature values while maintaining only the anatase structure, which may suggest defects on the TiO_2_ surface (e.g. formation of Ti^3+^ species). The application of microwaves was an efficient way to improve powder quality by enhancing crystallinity and decreasing processing times. Notably, the widely used microwave process borrowed from hydrothermal techniques often involves several stages, where heat treatment concludes the technological process^19,20^. However, microwave heating fundamentally differs from conventional processes such as oil-bath or hydrothermal heating regarding rapid reaction mechanisms, nucleation, and development of the formed material's nanostructure^21,22^. Therefore, to fully harness the potential of the microwave pathway, an innovative and comprehensive approach to the formation of nanomaterials is necessary.

Presently, an intriguing avenue in advancing the microwave route lies in utilizing the in situ process, a methodology hailed as "future-proof" by Kumar et al.^23^, enabling the continuous evolution of microwave phenomena. This innovative approach involves introducing a promoter into the reactor, which undergoes decomposition under the influence of rising temperatures during processing, thereby initiating the desired material's microwave-driven precipitation. The scientific literature reveals the successful application of hydrothermal in situ routes for synthesizing ZnO, notably employed as a component in concrete composites with heightened antibacterial activity^24^. Additionally, researchers have posited that in situ materials obtained through microwave means may serve as a versatile platform for the deposition of lanthanides^25^. The paramount advantage of in situ pathways is evident in their ability to facilitate one-step reactions, eliminating the need for intermediate product synthesis and preventing particle agglomeration while maintaining an advantageous particle size distribution^26,27^. These in situ routes have demonstrated remarkable efficiency by promoting crystalline growth and achieving desired crystallinity while also earning praise for their environmentally friendly attributes^28–30^. Significantly, this methodology allows precise control over the size and morphology of the formed materials by adjusting synthesis parameters, such as precursor concentration and the type and quantity of the precipitation agent^31^.

In this context, the application of an in situ microwave approach emerges as an innovative technique for synthesizing TiO_2_ NPs. This process involves using TiCl_4_ and NH_4_F as precursor materials. This unique synthesis pathway aims to produce multifunctional TiO_2_ nanoparticles with outstanding photocatalytic and photovoltaic performance. Additionally, our investigation delves into the influence of microwave treatment temperature on various physicochemical parameters. This technique enables the achievement of crystalline material even at temperatures below 100 °C. The TiO_2_ NPs synthesized through this approach exhibit significant effectiveness in removing formic acid, substantiating their practical applicability. Furthermore, our nanomaterials demonstrate impactful potential in dye-sensitized solar cells (DSSCs), providing an additional avenue for their utilization. This study underscores the paramount importance of environmental sustainability in materials science. It emphasizes that thoughtful management of the synthesis method makes it possible to progress toward developing multifunctional materials.

## Materials and method

### Materials

Titanium(IV) chloride (TiCl_4_, 97%, Merck, Germany), ammonium fluoride (NH_4_F, p.a., S Merck, Germany), formic acid (FA, 99%, Merck, Germany), ethylcellulose (p.a., Sigma-Aldrich, USA), α-terpineol (96%, Merck, Germany), 1-propyl-3-methyl-imidazole iodide (97%, Sigma-Aldrich, USA), 4-tert-butylpiridine (98%, Sigma-Aldrich, USA), potassium dihydrogen phosphate (p.a., Sigma-Aldrich, USA), acetonitrile (LC–MS ultra-pure, Honeywell, USA), acetic acid (p.a., POCh, Poland), anhydrous ethanol (99.8%, POCh, Poland), iodine (p., POCh, Poland), N3 dye (p., Ruthenizer 535, Solaronix, Switzerland), hexachloroplatinic acid hexahydrate (99.9%, Merck, Germany), hydrofluoric acid (40%, Suprapur, Merck, Germany), guanidine thiocyanate (97%, Fluka, Switzerland), ionomeric foil Meltonix (Solaronix, Switzerland), were used. All reagents were of analytical grade and used without any further purification. The water used in all experiments was deionized.

### Synthesis of TiO_2_ NPs

The synthesis of TiO_2_ NPs was achieved through the in situ microwave route. The precursor was titanium(IV) chloride, the solution of which was prepared in distilled water in an ice-water bath, according to the procedure described previously. The concentration of titanium(IV) chloride was adjusted to 1%. Subsequently, 1 g of ammonium fluoride was introduced into the 100 cm^3^ TiCl_4_ solution and stirred using an IKA magnetic stirrer (IKA Werke GmbH, Germany). The resulting clear solution was then transferred to a microwave reactor (CEM, Discover 2.0, USA) and heated with a maximum power of 300 W to temperatures ranging from 60 to 200 °C (in 20 °C increments). The heating process was conducted at various temperatures, where samples, e.g., T_60, were heated to 60 °C, and T_80 was heated to 80 °C. The solution was promptly cooled down to room temperature upon reaching the set temperature. Subsequent nanomaterials were obtained following the procedure above. Finally, the resulting nanomaterials were filtered, thoroughly washed, and dried at 60 °C for 6 h.

### Characterization of TiO_2_ NPs

The crystal structure analysis was done using a D8 Advance diffractometer (Bruker, Germany). The measured material was placed in a measuring cuvette and subjected to analysis with CuKα radiation (λ = 1.5406 Å) within the 2θ range of 20°–80° at a scan speed of 1°/min. XRD data were analyzed using Rietveld refinement method and applying the Fullprof program^32^. The crystallite size of the synthesized materials was determined using Williamson-Hall method^33^, represented by the equation:

1 $$\beta \cos \theta = \frac{K\lambda }{D} + 4\varepsilon \sin \theta$$

where *β*—is the line broadening at half the maximum intensity (FWHM), *θ*—is the Bragg angle, *K*—is a shape factor (0.891), *D*—size of crystallinity, *λ*—is the X-ray wavelength, and *ε*—lattice strain.

For the characterization of the porous structure, including BET surface area, pore volume, and pore size, a Quantachrome Autosorb iQ surface characterization analyzer (Quantachrome, USA) was employed. The Brunauer–Emmett–Teller (BET) method, based on low-temperature N_2_ sorption, was used for analysis. Surface area determination utilized the multipoint BET method with adsorption data in a relative pressure p/p_0_ range of 0.05–0.30.

Transmission electron microscopy (TEM) analysis was conducted using the FEI TECNAI G2 F20 electron microscope operating at 200 kV, featuring a Gatan CCD camera for high-resolution imaging. Specimens for high-resolution TEM (HR-TEM) analysis were prepared by sonication of a small amount of material in 2-propanol, followed by suspension onto a copper grid with a holey carbon film. Micrographs were captured after solvent evaporation, ensuring a comprehensive and statistically representative mapping of the studied materials.

X-ray Photoelectron Spectroscopy (XPS) experiments were recorded using a Specs UHV spectrometer (SPECS, Germany) with a charge neutralizer. The C 1*s* peak at 284.8 eV was a reference for rectifying the binding energies.

The light-absorption properties were measured through diffuse reflectance spectroscopy (DRS) in the 200–800 nm range. The bandgap energy of the samples was calculated from the (F(R)·E)^0.5^ against the E graph, where E is photon energy and F(R) is the Kubelka–Munk function proportional to the radiation's absorption. Thermo Scientific Evolution 220 spectrophotometer (Waltham, USA), equipped with a PIN-757 integrating sphere using BaSO_4_ as a reference, was employed for these measurements.

Photoluminescence (PL) measurements were conducted using a spectrofluorometer (Fluorolog version-3 Horiba, Japan) with a 450 W high-pressure xenon arc lamp as an excitation source. Photoluminescence excitation (λ = 320 nm) and emission spectra were acquired at room temperature with a spectral resolution of 2 nm and a slit width of 2 mm.

### Photocatalytic activity

In the standard procedure, a magnetically stirred 60 mL cylindrical quartz reactor was utilized and placed within a custom-made housing consisting of a black box mounted on an optical bench. The light source for irradiation was a convenient LED photosystem previously detailed elsewhere^34^, emitting ultraviolet light within the range of 360–410 nm. The entire setup was maintained at ambient temperature through a continuous air stream. All aqueous suspensions subjected to irradiation contained photocatalysts at a concentration of 0.1 g L^−1^. The photocatalysts, dispersed in pure water, underwent preliminary sonication in a Eurosonic, Model 22, apparatus for 30 min. Subsequently, the required volume of formic acid solution was added to achieve an initial formic acid concentration of 50 ppm. The suspension was then magnetically stirred in the dark for 15 min to reach the adsorption equilibrium of the substrate on the photocatalyst's surface before initiating irradiation. Stirring was maintained throughout the experimental runs. The lamp was switched on, and at various time intervals during the runs, 2 mL samples of the suspension were withdrawn from the reactor and centrifuged using an EBA-20 Hettich centrifuge (Hettich Group, Germany). The supernatant was analyzed for residual FA content through ion chromatography with conductivity detection, utilizing a Metrohm 761 Compact IC instrument (Metrohm AG, Switzerland), after calibration for formate ion concentration in the 0–50 ppm range.

Formic acid (FA) kinetics were assessed using a pseudo-first-order kinetic model. This model postulates that the degradation rate is directly proportional to the surface coverage (θ) of FA, expressed as follows:

2 $$r=\frac{dC}{dt}=k\theta =\frac{kK{C}_{0}}{1+K{C}_{0}+{K}_{s}{C}_{s}}$$

Here, *k* represents the reaction rate constant, 'θ' denotes the surface coverage by FA, *K* and *K*_*s*_ are the adsorption coefficients for FA and water, respectively, C_0_ stands for the initial concentration of formic acid, and C_s_ represents the concentration of water. The concentration of water, C_s_ remains nearly constant and is significantly higher than the concentration of formic acid. Consequently, we can express Eq. (3) in the following form:

3 $$ln\frac{{C}_{t}}{{C}_{0}}=-{k}_{1}t$$

In Eq. (3), *k*_*1*_ signifies the first-order rate constant, and *t* is the time of irradiation.

### Photovoltaic devices preparation

Working electrodes have been prepared by spreading titania paste on the FTO substrate using the doctor blade technique. The pastes containing investigated materials were obtained using the well-known literature method of mixing the titania powders with ethanol, alpha-terpineol, acetic acid, and ethylcellulose in the mass ratio 1:9.8:3.3:0.18:0.5, respectively^35^. After the paste deposition, working electrodes have been annealed at 450 °C in the air for 2 h. Cooled-down electrodes have been immersed in a 40 mM TiCl_4_ water solution for 1 h at 70 °C to prepare an electron recombination-preventing layer. Afterward, the electrodes were washed with distilled water and ethanol, dried, and again annealed at 450 °C for 1 h in the air. After cooling to approximately 80 °C, electrodes were immersed in 10^–4^ M N3 dye solution in absolute ethanol and left overnight. Counter electrodes have been prepared using our standard procedure by spreading H_2_PtCl_6_ ethanolic solution on FTO substrates followed by annealing at 450 °C in the air for 1 h^36^. Photovoltaic devices were assembled in the sandwich mode using a 25 µm thick ionomeric hot-melted foil as a spacer and sealing agent. Afterward, the liquid electrolyte, containing 0.6 M 1-propyl-3-methyl-imidazolium iodide, 0.03 M iodine, 0.1 M guanidine thiocyanate, and 0.5 M 4-tert-butylpiridine in acetonitrile, have been injected into the cell by two holes predrilled in the counter electrode and the final device seal have been made using an ionomeric foil and microscope cover slide. The typical active area of the investigated cells was 0.125 cm^2^. Additional working electrodes with an active area of about 2.5 cm^2^ have been prepared for the XRD and DRS measurements.

Photovoltaic parameters (I–V curves) measurements have been performed under AM 1.5G illumination using the Abet Sun 2000 solar simulator (Abet Technologies, USA). The light intensity was calibrated to 1 sun using a ReRa Solutions reference cell with a KG5 filter (ReRa Solutions, The Netherlands). Electrochemical impedance spectroscopy (EIS) has been performed in the 1 sun illumination over a frequency range from 0.1 Hz to 100 kHz at VOC bias potential, with sinusoidal VAC = 10 mV. The I–V curves and EIS spectra have been registered on a Gamry Interface 1010 E potentiostat–galvanostat (Gamry Instruments, USA). EIS data have been fitted using ZView 3.2 software. Incident photon to current conversion efficiency (IPCE) was investigated on Bentham PVE300 EQE/IPCE apparatus (Bentham Instruments Ltd., UK) using a chopped mode with an external light bias.

## Results and discussion

### Physicochemical characterization of synthesized TiO_2_ NPs

**Figure 1:**
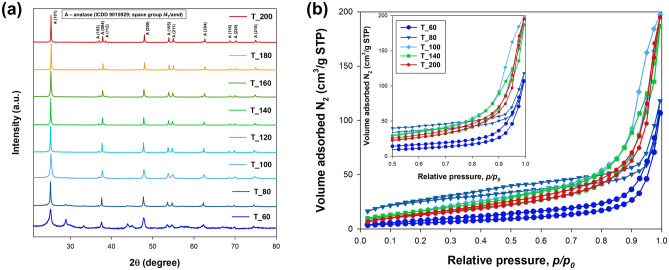
Results of (**a**) XRD patterns, and (**b**) low-temperature N_2_ sorption isotherms for TiO_2_ NPs fabricated by microwave in situ route.

In the initial phase of the physicochemical analysis, X-ray diffraction (XRD) was conducted to determine the capability of proposed microwave in situ pathways for crystal growth and achieving the desired crystallinity. The resulting XRD patterns are illustrated in Fig. 1. The distinct peaks identified in the XRD patterns of the TiO_2_ nanomaterials, occurring at 2θ values of 25.28°, 36.9°, 37.8°, 47.9°, 53.8°, 55°, 62.6°, 68.7°, 70°, and 75.05°, closely correspond to the characteristic peaks of the anatase crystalline structure (as per crystallographic database card no. 9009086) in space group *I*_*41*_*/amd* (no. 141)^37–39^. Furthermore, the XRD analysis disclosed the presence of crystalline planes, specifically (101), (103), (004), (200), (105), (211), (213), (116), (220), and (215) in the analyzed nanomaterials^40^. It should be emphasized, however, that in the case of materials obtained within the temperature range of 60–100 °C, additional diffraction peaks were observed, suggesting the presence of other crystalline phases, particularly titanium fluoride, with its characteristic peak observed at 2θ = 28.6°^41,42^. Nevertheless, irrespective of the temperature applied in the microwave process, no discernible peaks characteristic of other titanium dioxide phases, such as rutile and brookite, were identified. This observation underscores that the proposed methodology exclusively promotes the growth of crystalline anatase.

Rietveld refinement was performed to obtain a thorough insight into the crystallinity of the acquired materials, and the corresponding results are summarized in Figure S1 of the Supplementary Materials. The determined lattice parameters and average size of crystallites are detailed in Table S1 (see Supplementary Materials). Figure S2 compares the anatase peak location at 2θ of about 25°. Analysis of the collected data reveals a noticeable shift in the anatase peak towards larger values with the increasing microwave processing temperature. This shift likely indicates a reduction in the lattice distance (*d*), causing the peak to shift towards higher 2θ values. As per the Bragg equation, the decrease in the distance between crystal planes results in an increased 2θ angle, implying a contraction in the crystal dimensions along a specific direction^43^. This observation is supported by the determined parameters of the anatase crystal lattice. However, it is crucial to emphasize that the shift in the peak towards higher 2θ values may also be attributed to stresses within the crystal lattice. Stress-induced changes can affect the separation between crystal planes, thereby influencing X-ray diffraction patterns. In the analyzed samples, stress–strain (ε) values were quantified, revealing a proportional increase with the rise in microwave processing temperature^44^. The above underscores the dynamic nature of the in situ microwave process, leading to heightened stresses within the crystal structure as the process temperature increases. Moreover, the analysis of the determined parameters indicates that an increase in microwave processing temperature leads to crystalline growth, a phenomenon we have previously validated in our earlier studies. However, the obtained XRD data show that the in situ microwave process causes the crystallization of anatase with surface defects, linked to the contraction of the crystal lattice in the *c*-direction and the presence of stress–strain.

The N_2_ isotherms depicted in Fig. 1b exhibit characteristic features of type IV shape, indicating reversible mono- and multilayer adsorption in the lower range of *p/p*_0_, followed by a hysteresis loop at higher pressure values^45^. Across all synthesized materials, a discernible reduction in the adsorbed volume is evident with an increase in microwave temperature, particularly in the lower section of the isotherm, aligning with the observed improvement in material crystallinity. Furthermore, two specific types of hysteresis loops were identified. Materials synthesized at 60–80 °C and 160–200 °C displayed an H3 hysteresis loop, indicating aggregates of plate-like particles giving rise to slit-shaped pores^46,47^. In contrast, materials obtained at temperatures of 100–140 °C exhibited an H1 hysteresis loop, often associated with porous materials consisting of agglomerates or compacts of approximately uniform spheres in a fairly regular array, resulting in narrow pore size distributions^45^. These distinctions are closely tied to the crystallinity of the materials. At a temperature around 100 °C, the transformation of amorphous TiO_2_ or titanium fluoride into nanocrystalline anatase occurs, forming agglomerates composed of TiO_2_ nanoparticles. This transformation significantly contributes to the observed augmentation in specific surface area and total pore volume. With a further increase in temperature (above 140 °C), notable crystalline growth occurs, leading to the formation of tetragonal TiO_2_ nanoparticles. This results in decreased specific surface area and the emergence of slit-shaped pores between nanocrystalline anatase particles. A comprehensive set of sorption measurements, encompassing calculated BET surface area, total pore volume, and average pore size, is presented in Table S1 in Supplementary Materials.

**Figure 2:**
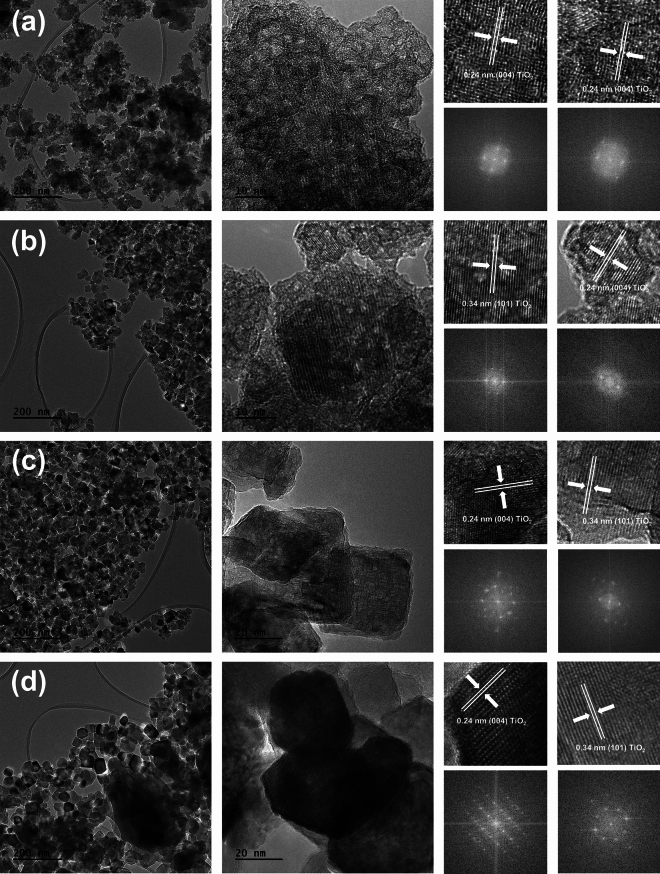
The TEM, HR-TEM, and FFT images for: (**a**) T_60, (**b**) T_100, (**c**) T_140, (**d**) T_200.

For a comprehensive investigation of TiO_2_ NPs, both TEM, HRTEM and FFT analysis were carried out. The outcomes are illustrated in Fig. 2.

In the case of the T_60 material (Fig. 2a), a distinct structure is evident in the high-contrast image, indicating a high contribution of the amorphous phase. High-resolution imaging shows crystallographic spacings indicative of partial crystallization, consistent with XRD data. Additionally, only single spots are observed in the obtained FFT images, which may indicate poor crystallinity of the sample and is consistent with previous reports by Bielan et al.^48^ and Dozzi et al.^49,50^. Increasing the microwave treatment temperature to 100 °C (sample T_100, Fig. 2b) results in an elevated share of the crystalline phase. Additionally, single crystalline particles, including cubic ones, are observable in lower magnification images. High-resolution images confirm the presence of lattice spacings characteristic of anatase. The number of spots in the FFT image indicates that the rapid microwave crystallization resulted in the growth of TiO_2_ crystals in different directions, which is confirmed by the varied morphology forms and wide particle size distribution^51^. For sample T_140 (Fig. 2c), only nanocrystalline particles are observed, without any amorphous areas, indicating that 140 °C is the temperature limit, allowing for nearly complete crystallization of anatase in the in situ microwave procedure. In the detailed examination of the T_140 sample, an ordered morphology was revealed, characterized by the presence of octahedral anatase TiO_2_ bipyramids with well-exposed {001}/{101} facets, aligning with previous reports by Kubiak et al.^52^. This morphological evolution is attributed primarily to two factors: termination by fluoride ions, promoting crystal growth in specific directions, and the extension of the microwave process. Fluoride ion termination was found to stabilize (001) surfaces over (101) due to the balancing of O–O/O–F repulsions and Ti–O/Ti–F attractions, stabilizing Ti and O atoms on the surface. Further raising the microwave treatment temperature to 200 °C (sample T_200, Fig. 2d) leads to crystal growth, increasing the average particle size to 40–60 nm. Additionally, a visible change in the shape of the obtained nanoparticles is noted. In contrast to the T_140 material, the nanoparticles obtained for T_200 are characterized by a predominance of octahedral shape. These considerations clearly confirm previous XRD results, demonstrating that the in situ microwave processing temperature decisively influences the morphology of the obtained materials.

**Figure 3:**
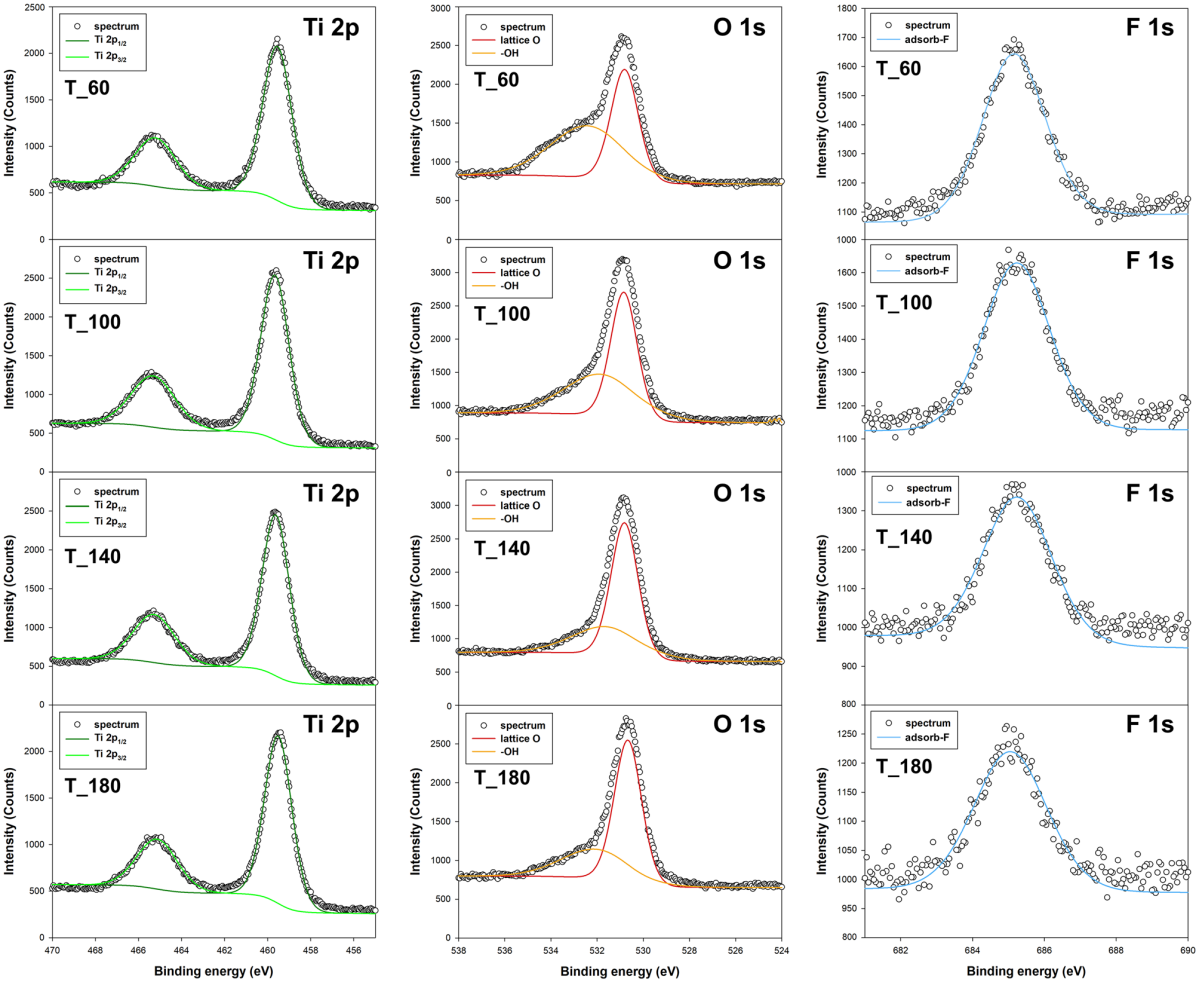
XPS spectra of Ti 2*p*, O 1*s*, and F 1*s* for TiO_2_ NPs.

X-ray Photoelectron Spectroscopy analysis was employed to investigate the surface composition and oxidation states of elements in the TiO_2_ nanoparticles. The examination of XPS data substantiated the presence of three crucial elements integral to the synthesis process, namely Ti, O, and F. The high-resolution XPS spectra are presented in Fig. 3, and Table S2 in the Supplementary Materials provides a comprehensive summary of the atomic percentages of each element. Noteworthy is the observed variation in fluoride atomic content across the analyzed systems, demonstrating a decline as the temperature of microwave treatment for the samples increases.

The Ti 2*p* region reveals two distinct peaks at 459.3 eV and 465 eV, attributed to Ti 2*p*_3/2_ and Ti 2*p*_1/2_, respectively. The 5.7 eV separation between Ti 2*p*_1/2_ and Ti 2*p*_3/2_ signifies a standard Ti^4+^ state^53–55^. In the O 1*s* region, TiO_2_ spectra exhibit a double peak that can be resolved into two oxygen atom types: lattice oxygen (530.5 eV) and adsorbed surface −OH groups (532.1 eV), with a spacing consistent with literature values, such as the 1.5 eV difference between lattice O^2−^ and −OH^56,57^. The F 1*s* region displays a peak at 685 eV, corresponding to F atoms adsorbed on TiO_2_. This F 1*s* peak originates from surface fluoride formed by ligand exchange between F^−^ and surface hydroxyl groups on TiO_2_. The F 1*s* peak is precisely located at 685 eV, and no evidence of F^−^ ions in the lattice was detected, aligning with existing literature^14,58^.

**Figure 4:**
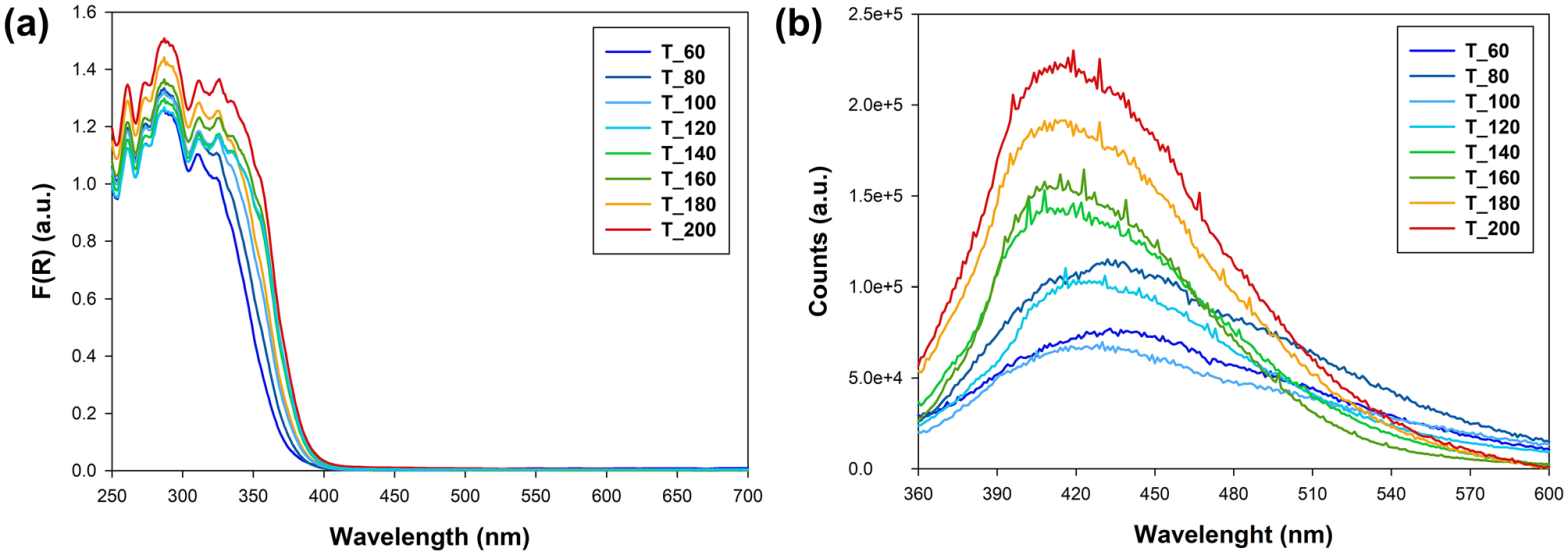
The (**a**) DRS and (**b**) emission spectra for TiO_2_ NPs.

Analysis of DRS data reveals the presence of a well-established absorption band in the 250–400 nm range for the synthesized TiO_2_ nanomaterials (Fig. 4a). It is noteworthy that an increase in the microwave processing temperature leads to a gradual enhancement of absorbance, particularly towards longer wavelengths. This phenomenon can be attributed to alterations in the quantity of O *2p* bonding orbitals, specifically those within the Ti_3_O plane^51^. The energy bandgap of the synthesized samples was determined using the Kubelka–Munk equation. Tauc plots for selected materials are shown in Figure S3 in the Supplementary Materials. An absorption peak at around 3.0–3.1 eV is consistently observed across all samples, denoting electron transfer from the valence band to the conductivity band in the pure anatase structure, aligning with existing literature. It is essential to highlight that introducing fluorine ions during the synthesis of TiO_2_ nanoparticles often leads to the formation of surface Ti–F bonds, absorbing light around 500 nm, as reported by Dudziak et al.^59^. However, this effect is not observed in our materials, indicating the absence of Ti-F bonds on the surface of the obtained anatase nanocrystals. Furthermore, the lack of a band gap shift is correlated with the absence of surface defects, as confirmed by the XPS results.

The observed photoluminescence emission (Fig. 4b) primarily results from the recombination of excited electrons and holes, offering crucial insights into carrier separation efficiency in photo-induced processes. Across all examined materials, a consistently noted broad luminescence band around 450 nm was observed. In contrast, the typical literature on TiO_2_ photoluminescence spectra highlights two primary emission peaks at approximately 396 and 462 nm, corresponding to energies of 3.13 and 2.68 eV, respectively^40,60^. However, broader luminescence bands encompassing these narrow peaks have also been documented, as reported by Meng et al.^61^. These peaks are attributed to bandgap transition emission with light energy roughly equal to the anatase bandgap energy (387.5 nm) and an emission signal arising from the charge-transfer transition from Ti^3+^ to oxygen anions in a TiO_6_^8−^ complex^62,63^.

Notably, the obtained PL spectra can be categorized into two groups based on the synthesis temperature. In the microwave processing temperature range of 60–120 °C, lower luminescence efficiency and a peak shift towards longer wavelengths, peaking at 430 nm, were observed. Conversely, for materials synthesized at temperatures ranging from 140 to 200 °C, an increase in luminescence efficiency was evident, with a peak at approximately 420 nm. These findings suggest that elevating the in situ microwave processing temperature enhances luminescence and increases charge carrier recombination.

### Application of TiO_2_ NPs

#### Photocatalytic Activity

**Figure 5:**
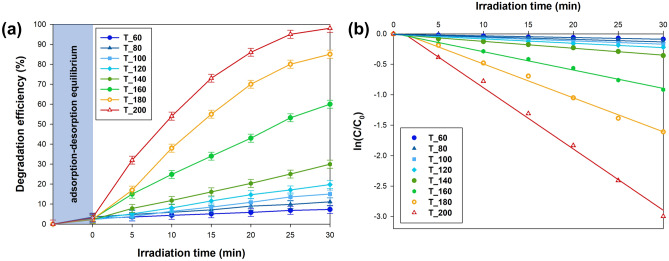
(**a**) Photocatalytic degradation efficiency and (**b**) first-order plots for the photooxidation of formic acid.

One crucial aspect of the research was assessing the photooxidation capabilities of the TiO_2_ NPs in the formic acid degradation. This evaluation was conducted utilizing a convenient LED photoreactor, and the findings are illustrated in Fig. 5.

The influence of microwave treatment temperature on the photocatalytic activity of TiO_2_ is evident (Fig. 5a). The T_60 sample exhibited the lowest degradation efficiency due to its reduced crystallinity, displaying only a 25% maximum efficiency within the 60–140 °C temperature range. However, increasing the temperature beyond 140 °C significantly enhanced the pollutant removal efficiency, with the TiO_2__200 sample achieving the highest degradation rate of 99% after 30 min. Interestingly, the T_200 material showed the highest charge carrier recombination, as indicated by photoluminescence data, although this did not negatively impact formic acid degradation. The superior performance of the TiO_2__200 sample is primarily attributed to its highly crystalline anatase structure with minimal surface defects, as revealed by X-ray diffraction (XRD) analysis. These minimal defects reduce electron–hole recombination, thereby enhancing photocatalytic efficiency. Additionally, the morphology of this sample, characterized by uniform octahedral shapes with well-defined facets, provides numerous active sites for the photocatalytic reaction. TEM analyses further supported this beneficial morphology, showing a predominant crystalline phase with larger particle sizes that promote improved charge carrier dynamics. Moreover, DRS measurements indicated a slight blue shift in the absorption edge for the TiO_2__200 sample, which enhances UV light utilization and potentially extends the light absorption spectrum. Collectively, these physicochemical properties contribute to the enhanced photocatalytic activity of the TiO_2__200 sample, clearly demonstrating the impact of synthesis conditions on material properties.

The sorption process was intentionally excluded from the calculations to establish the rate constant parameter, as shown in Fig. 5b. Using Eqs. (2) and (3), the apparent values of parameter *k*_*1*_ for each catalyst were determined by analyzing the slope of the ln C_t_/C_0_ vs. time plot. The computed values are detailed in Table S3 and available in the Supplementary Materials for reference and comparison. The outcomes underscore the significant impact of microwave treatment temperature on the photooxidative capacity of the synthesized materials. Among the studied materials, the T_200 material exhibited the highest reaction rate constant at 0.095 min^−1^. Despite increased charge carrier recombination, as evident in the photoluminescence spectra, the reaction rate constantly rose with elevated microwave treatment temperatures. The above further proves that the formic acid degradation process with TiO_2_ nanoparticles is not solely contingent on charge carrier recombination but is intricately linked to physicochemical parameters such as crystal structure and morphology.

Nanostructured titanium dioxide's synthesis methods and physicochemical properties significantly influence its photocatalytic performance. For instance, research conducted by Dorosheva et al.^65^ focused on preparing nanostructured TiO_2_ via a sol–gel process, resulting in photocatalytically active materials for organic reactions under visible light exposure. The photocatalytic activity of these TiO_2_ nanoparticles was notably enhanced by controlling the crystallite size, ranging from 10 to 85 nm. This size modification was achieved through annealing processes, inducing the transformation of the material's phase from amorphous to anatase under treatment in a hydrogen atmosphere at specific temperatures. Similarly, the study conducted by Parida et al.^66^ on zinc oxide revealed significant influences of physicochemical properties such as surface area, surface acidity, and crystallite sizes of ZnO particles on photocatalytic activity towards the oxidation of 4-nitrophenol and the reduction of Cr(VI). ZnO samples prepared by microwave irradiation and calcined at 300 °C exhibited the highest surface area, acid sites, and the lowest crystallite sizes, leading to superior activity in photocatalytic reactions, this underscores the pivotal role of nanoparticle morphology in influencing photocatalytic efficiency.

These findings underscore the crucial role of the morphological characteristics of photocatalysts in determining their efficiency^67,68^. By adjusting synthesis conditions and post-synthesis treatments, researchers can tailor nanoparticles' crystal structure, surface properties, and morphology to optimize their photocatalytic performance. The correlation between nanoparticle morphology and photocatalytic activity highlights the importance of detailed physicochemical characterization in developing efficient photocatalytic materials.

Similar conclusions were drawn based on obtained SEM images (see Figure S4 in the Supplementary Materials). An increase in microwave processing temperature resulted in a more uniform particle size distribution, facilitating the production of material with uniform morphology, which critically impacted the efficiency achieved in the formic acid degradation process.

Table 1 shows the current state of knowledge in the elimination of formic acid.

**Table 1:** Summary of literature data on the photodegradation of formic acid.

Sample	Formic acid concentration (mg/dm^3^)	Amount of photocatalyst (g/dm^3^)	Degradation efficiency (%)	Irradiation time (min)	Type and power of the light source	Literature
T_200	50	1	98	30	360–410 nm nm UV-LED	This work
TiO_2_	50	0.25	50	120	340 nm (LED)	^69^
HNT-1% PHF-400	200	1	99	60	125 W Hg lamp	^70^
TNCA2	50	1	99	40	PL-L (18 W) lamp	^71^
0.8 wt% Sr-NT	100	1	99	90	PL-L (18 W) lamp	^72^
TiO_2_	50	1	99	120	125 W mercury lamp	^73^
EST00-80A	25	1	99	20	Solarium HB175 lamp (60 W)	^74^

The T_200 and T_180 photocatalysts were chosen for the reusability test. Five consecutive cycles of formic acid photodegradation were conducted to assess photocatalytic reusability, as depicted in Figure S5 in Supplementary Materials. After each cycle, filtration separated the photocatalyst from the reaction suspension. Subsequently, the separated photocatalyst was reused without any further treatment. The photocatalytic degradation efficiency decreased by approximately 5% after the fifth cycle compared to the first. This slight decrease in activity after each irradiation cycle could be attributed to photocatalyst losses during the separation process.

#### Photovoltaic properties

Studied materials have also been tested as semiconductor layers in DSSCs. It should be emphasized that during the working electrode preparation, the materials were annealed at 450 °C (the detailed procedures described in the Experimental section of this manuscript), significantly influencing their structure. However, their properties were still strongly related to the nature of the bare samples. The XRD analysis of prepared electrodes, see Figure S6 in Supplementary materials, shows that all the materials present well-formed anatase structures with no post-synthetic fluorine moieties residues. The additional reflexes observed on each diffractogram originated from the SnO (JPDS no. 2-1337), the conductive layer of FTO substrates. The reduced intensities of the main anatase reflexes observed for the samples synthesized at lower temperatures compared to those obtained at higher ones prove the influence of primary preparation temperature on the material's properties even after high-temperature post-treatment. The electron properties of the investigated materials were also changed after annealing during the electrode preparation. As the main DRS spectra shape has been preserved (see Figure S7 in Supplementary materials), the hypochromic effect of the absorption edges of particular materials is observed (see Table 2). Still, higher E_g_ values are observed for the materials prepared at lower synthesis temperatures. Several authors have previously described such effects—the preservation of some properties tendencies of the TiO_2_ materials originally prepared in different temperatures^75,76^. This type of behavior may be understood as a kind of synthesis-conditions-fingerprint of the material. The above observations lead to the expectation of significant differences in investigated materials' photovoltaic parameters.

**Table 2:** Photovoltaic properties of assembled cells and E_g_ values of prepared semiconducting layers.

	J_SC_ (mA/cm^2^)	V_OC_ (mV)	FF (%)	η (%)	IPCE (%)	E_g_ (eV)
T_60	3.96	679	63.3	1.70	23.4	3.20
T_80	7.11	678	52.7	2.54	28.1	3.20
T_100	12.2	663	66.1	5.33	48.5	3.20
T_120	8.72	695	61,8	3,75	30.1	3.19
T_140	9.84	669	63.9	4.21	43.4	3.18
T_160	11.0	670	66.5	4.91	45.4	3.15
T_180	9.50	681	65.8	4.25	41.1	3.18
T_200	10.8	693	61.1	4.57	43.9	3.18

**Figure 6:**
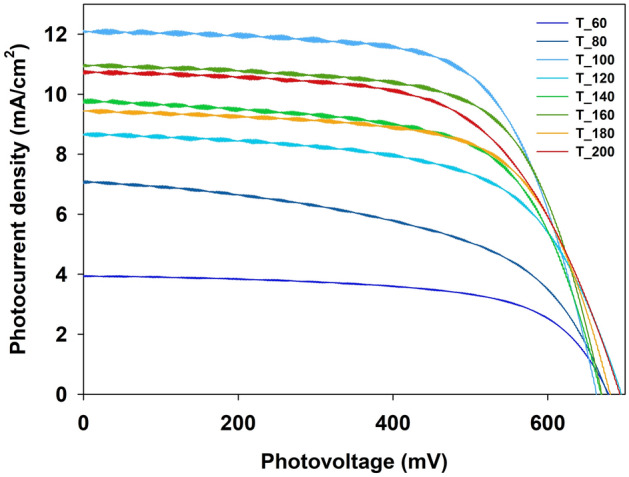
J–V curves registered for DSSCs studied.

Assembled DSSC devices have been characterized with J–V curve measurements under simulated solar light, and the results are presented in Fig. 6 and Table 2. The performances of the DSSCs present somewhat different tendencies than those observed in our photocatalytic results. Surprisingly, the best photoconversion efficiency (η) has been observed for the T_100 device. The materials prepared in lower temperatures, T_60 and T_80, were the least efficient as electrode materials in DSSCs, similar to photocatalysis. Moreover, following our photocatalytic results, the materials obtained at 140 °C and above were more efficient. The more profound analysis of the photovoltaic parameters shows that the reason for the T-100 cell's best efficiency is the outstanding photocurrent density (J_SC_) of 12.2 mA/cm^2^, 10% better than the second-best registered for T_160 material (11.0 mA/cm^2^) and over three times better than the worst result registered for T_60 cell (3.96 mA/cm^2^). Such results suggest that the T_100 working electrodes have relatively good electron transportation properties and/or poor electron recombination process efficiency. The ease in electron transportation may also be the reason for such good T-100 cell efficiency, even though the open circuit potential (V_OC_) value, which is the driving force of electron transportation in the device^77^, is the lowest among investigated cells. At this stage, the fill factor (FF) values, mainly understood as energy loss related to inherent resistance in the photovoltaic device^78,79^, seem worth considering. In this case, the FF values do not vary as much as the other photovoltaic parameters of the presented cells, but still, the result registered for the T_100 cell is one of the best, yielding only the one obtained for the T_160 cell. The relatively high FF value may suggest that the internal resistances in the T_100 cell are most probably optimal for good electron transportation and to avoid the recombination effects.

Electrochemical impedance spectroscopic measurements were performed for deeper insight into the resistance properties of investigated cells. The Nyquist plots obtained for investigated cells are presented in Figure S8 in Supplementary materials, and the calculated values of the resistances (R_1_, R_2_, and R_3_, obtained by fitting the experimental results) and estimated electron lifetimes are collected in Table S4 in Supplementary materials. As R_1_ and R_2_ values, which are the serial resistance of the FTO substrate or wires connection and the charge transfer resistance at the counter electrode/electrolyte interface, respectively, are mainly considered to have no significant influence on DSSCs performance^80,81^, we focus on the R_3_ value interpretation. The R_3_ value, the second semicircle on the Nyquist plot, represents resistances in the TiO_2_/dye/electrolyte interface and directly influences the cell performance. One may see that the R_3_ values are the almost exact representation of the J_SC_ values according to dependence; the lower the R_3,_ the higher the J_SC_. This observation strongly supports our abovementioned hypotheses that the ease in electron transportation through T_100 electrode material is the most probable reason for its outstanding performance. On the other hand, the analysis of estimated electron lifetimes (τ) suggests that the stability of the electrons injected into the T_100 semiconductor is not as unusual when compared to the other materials tested. The latter observation may suggest that the lower efficiency of PL does not simply translate to the cell performance.

The IPCE measurements were performed to determine the efficiency of the conversion incident photon into the cell's internal photocurrent, and the results obtained are presented in Figure S9 in Supplementary Materials and Table 2. In short, the shape of the IPCE curves approximately reflects the sensitizer's (N3 dye) absorption spectrum. IPCE values determined about the N3 dye adsorption maximum (~ 540 nm) refer to the J_SC_ values supporting the above discussion about the crucial role of the ease of electron injection and transportation in the performance of presented cells^81,82^.

## Conclusions

In this study, TiO_2_ nanomaterials were synthesized using an innovative microwave in situ method, presenting a novel approach for their preparation. The investigation highlighted the significant impact of microwave treatment temperature on key physicochemical properties, such as crystal structure, morphology, and surface area development. Importantly, higher microwave processing temperatures were observed to stimulate crystallite growth, as evidenced by HR-TEM images, thereby influencing the content of the crystalline phase. The precise control over material properties achieved through in situ microwave processing temperature makes the resulting TiO_2_ nanoparticles suitable for diverse applications.

Notably, TiO_2_ nanomaterials synthesized at elevated microwave treatment temperatures exhibited improved performance in formic acid photo-oxidation. Despite an increase in luminescence intensity, indicating charge carrier recombination, the photocatalytic activity showed a positive correlation with the rise in microwave treatment temperature. This observation suggests that the photodegradation process of formic acid is not dependent on the rate of charge carrier recombination.

Moreover, the synthesized materials were employed in DSSC, with the T_100 material demonstrating the highest efficiency. This emphasizes the significance of customized material properties in achieving enhanced activity and reduced energy consumption during synthesis by utilizing lower process temperatures. Such an approach represents a crucial step towards incorporating sustainability into materials science, showcasing a conscientious effort to address environmental concerns in material synthesis.

### Supplementary Information

Supplementary Information.

## Data Availability

The data that support the findings of this research are available from the corresponding author upon reasonable request.
