# Supplementary material for: Innovative microwave in situ approach for crystallizing TiO2 nanoparticles with enhanced activity in photocatalytic and photovoltaic applications
Source: Sci Rep. 2024 Jun 1;14:12617. doi: 10.1038/s41598-024-63614-7 (PMC11144198; doi:10.1038/s41598-024-63614-7)
Supplement: Supplementary file 1 — Supplementary Information. [file 41598_2024_63614_MOESM1_ESM.docx]

**Innovative microwave *in situ* approach for crystallizing TiO_2_ nanoparticles with enhanced activity in photocatalytic and photovoltaic applications**

Adam Kubiak^*^, Maciej Zalas, Michał Cegłowski

*Adam Mickiewicz University, Poznan, Faculty of Chemistry, Uniwersytetu Poznanskiego 8, PL-61614 Poznan, Poland*

**Corresponding author: adam.kubiak@amu.edu.pl; Tel.: +48 61 829 17 21*


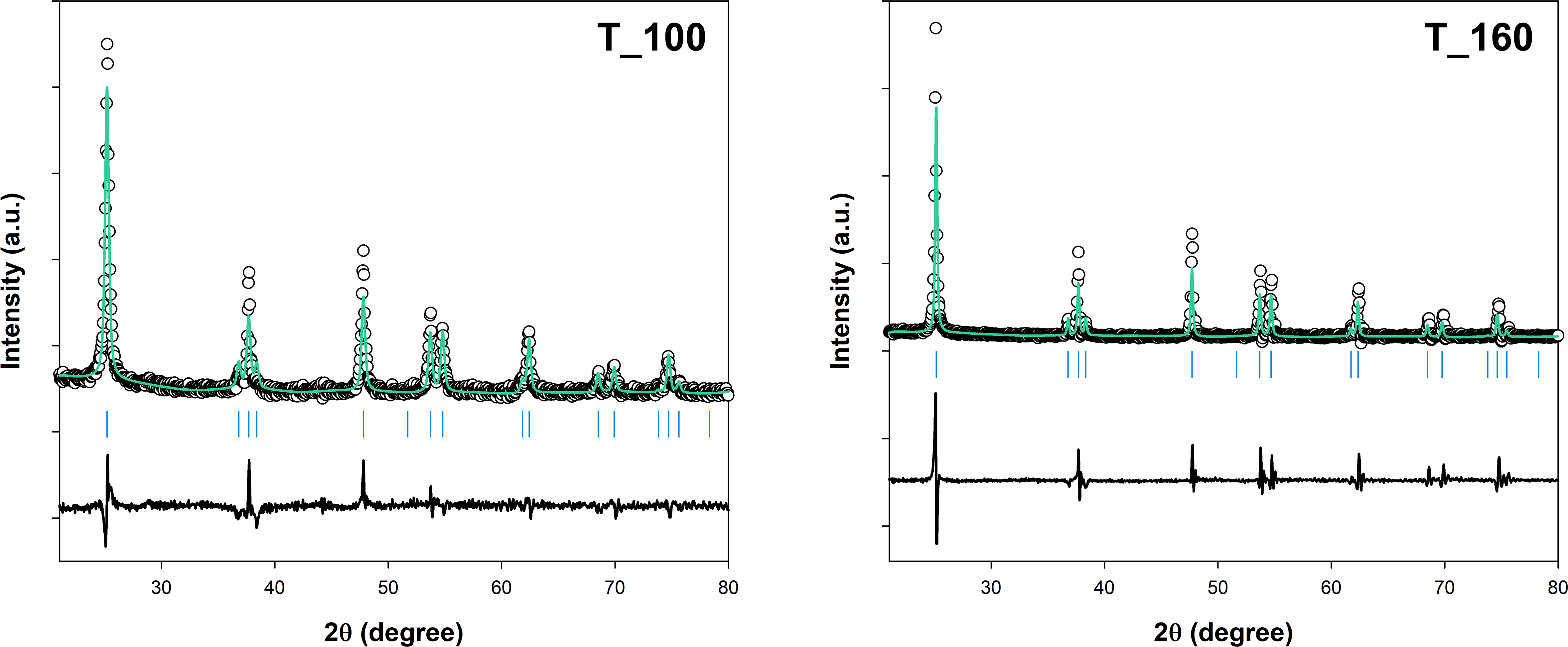


**Figure S1.** Example of Rietveld refinement for selected TiO_2_ materials.


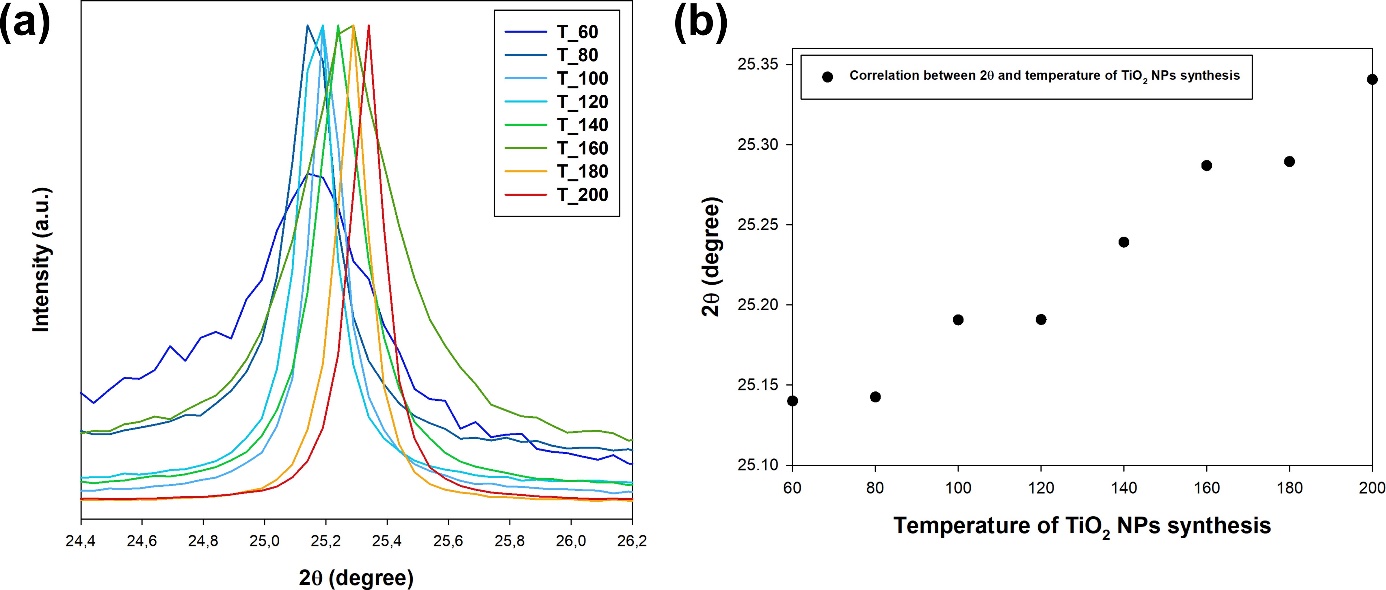


**Figure S2.** (a) XRD patterns in the 2θ range of 24.4-26.2 for TiO_2_ NPs fabricated by the microwave *in situ* route, and (b) the position of the anatase peaks (2θ = 25.2°) depending on the temperature of TiO_2_ NPs synthesis.


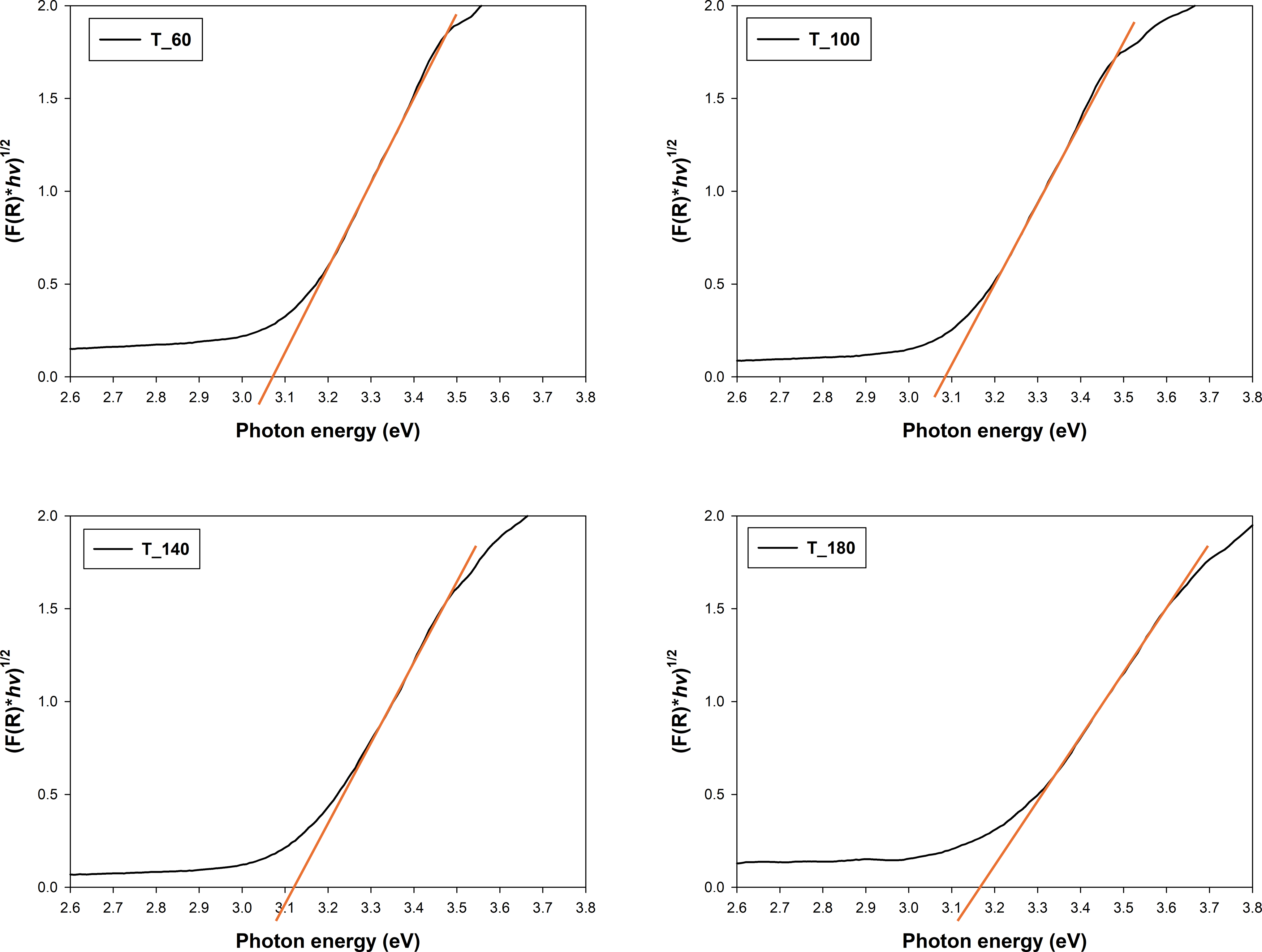


**Figure S3.** The Tauc plot for selected TiO_2_ NPs materials.

**Table S1.** X-ray diffraction, low-temperature N_2_ sorption, and diffuse reflectance spectroscopy results for synthesized TiO_2_ NPs.

| Sample | *D* (nm) | Lattice parameters | | ε | Porous structure parameters | | | Energy band gap | |  |
| --- | --- | --- | --- | --- | --- | --- | --- | --- | --- | --- |
|  |  | **anatase** | |  | **A_BET_ (m^2^g^-1^)** | **V_p_ (cm^3^g^-1^)** | **Sp (nm)** | | **E_g_ (eV)** | |
|  |  | ***a* (Å)** | ***c* (Å)** |  |  |  |  |  |  |  |
| T_60 | 8.0 | 3.8191 | 9.5962 | 0.003 | 76 | 0.450 | 3.7 | | 3.05 | |
| T_80 | 9.8 | 3.8196 | 9.5862 | 0.004 | 87 | 0.438 | 4.5 | | 3.05 | |
| T_100 | 11.8 | 3.8195 | 9.5559 | 0.006 | 101 | 0.500 | 5.4 | | 3.05 | |
| T_120 | 18.1 | 3.8179 | 9.5388 | 0.006 | 78 | 0.385 | 4.1 | | 3.10 | |
| T_140 | 20.1 | 3.8020 | 9.5382 | 0.008 | 71 | 0.307 | 3.7 | | 3.10 | |
| T_160 | 21.3 | 3.7991 | 9.5174 | 0.010 | 67 | 0.297 | 3.1 | | 3.15 | |
| T_180 | 25.1 | 3.7935 | 9.5141 | 0.011 | 68 | 0.175 | 2.9 | | 3.15 | |
| T_200 | 26.4 | 3.7902 | 9.5087 | 0.013 | 61 | 0.165 | 2.8 | | 3.15 | |

**Table S2.** XPS analysis of Ti, O, F content (at.%) for the selected TiO_2_ materials.

| Sample | Content (at.%) | | |
| --- | --- | --- | --- |
|  | **Ti 2p** | **O 1s** | **F 1s** |
| **T_60** | 25.4 | 65.1 | 9.5 |
| **T_100** | 26.0 | 66.4 | 7.6 |
| **T_140** | 29.8 | 64.6 | 5.4 |
| **T_180** | 32.9 | 63.9 | 3.2 |

**Table S3.** The calculated kinetic parameters for the photochemical degradation of formic acid.

| Sample | k_1_ (1/min) | (R^2^) |
| --- | --- | --- |
| T_60 | 0.0030 | 0.993 |
| T_80 | 0.0045 | 0.994 |
| T_100 | 0.0057 | 0.996 |
| T_120 | 0.0077 | 0.996 |
| T_140 | 0.0118 | 0.997 |
| T_160 | 0.0297 | 0.998 |
| T_180 | 0.0528 | 0.998 |
| T_200 | 0.0950 | 0.996 |


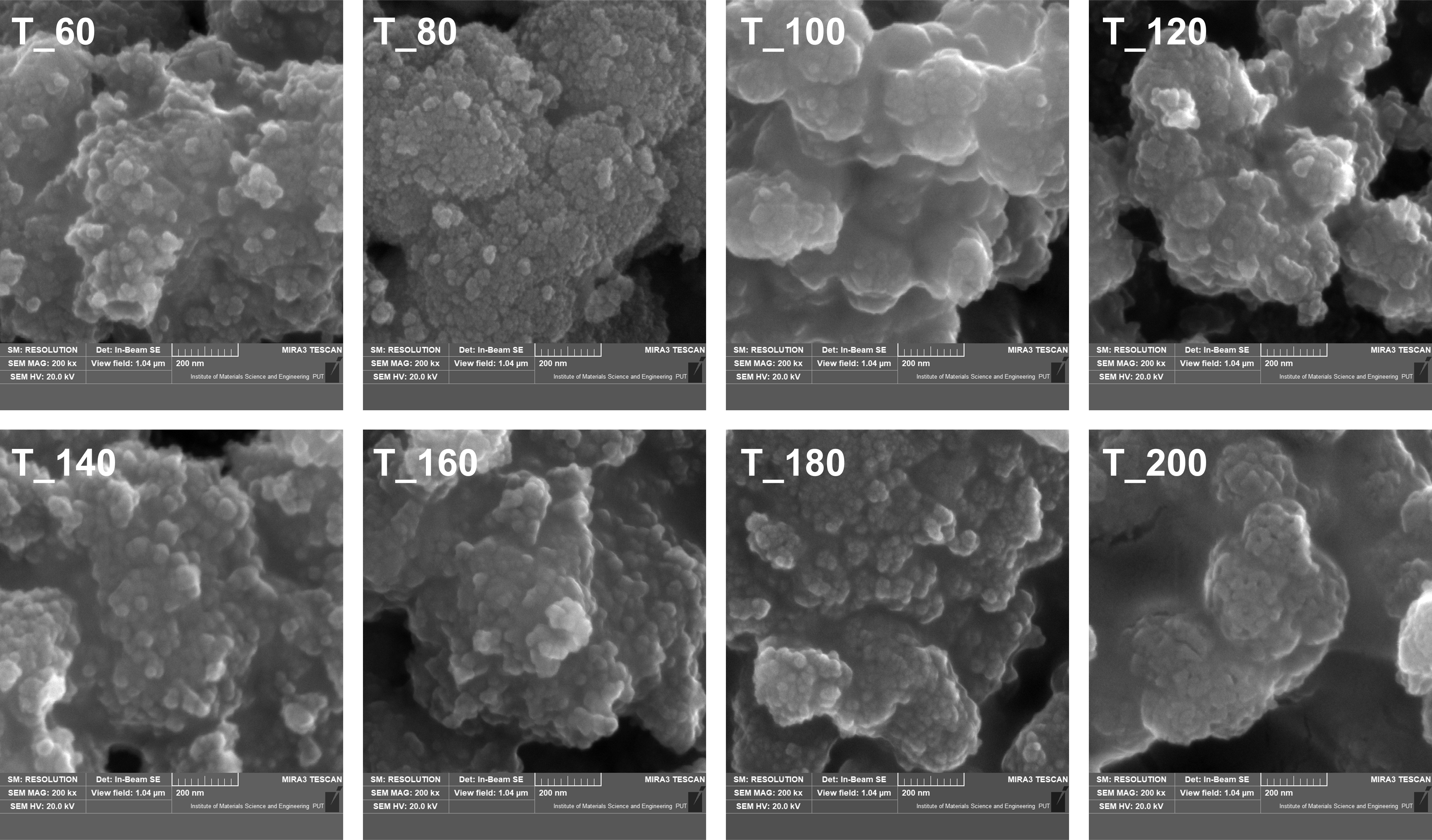


**Figure S4.** The SEM images for synthesized TiO_2_ NPs.


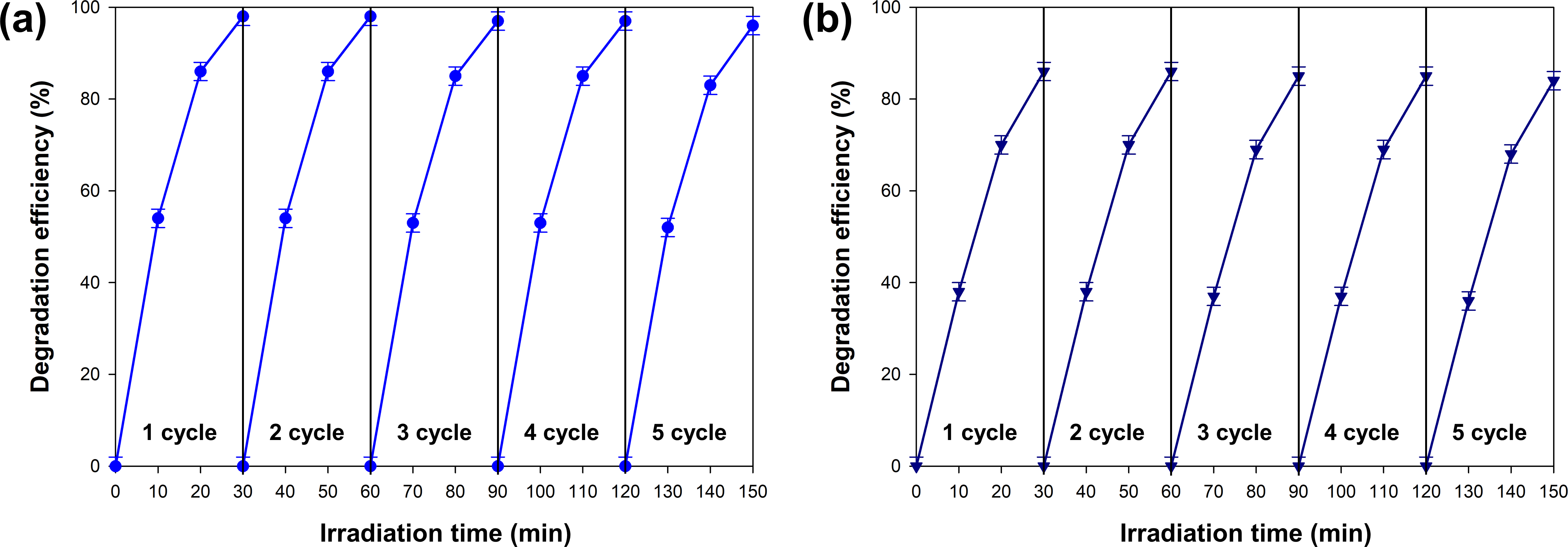


**Figure S5.** Efficiency of formic acid photodegradation in the presence of (a) T_200 and (b) T_180 photocatalysts measured over five successive cycles.


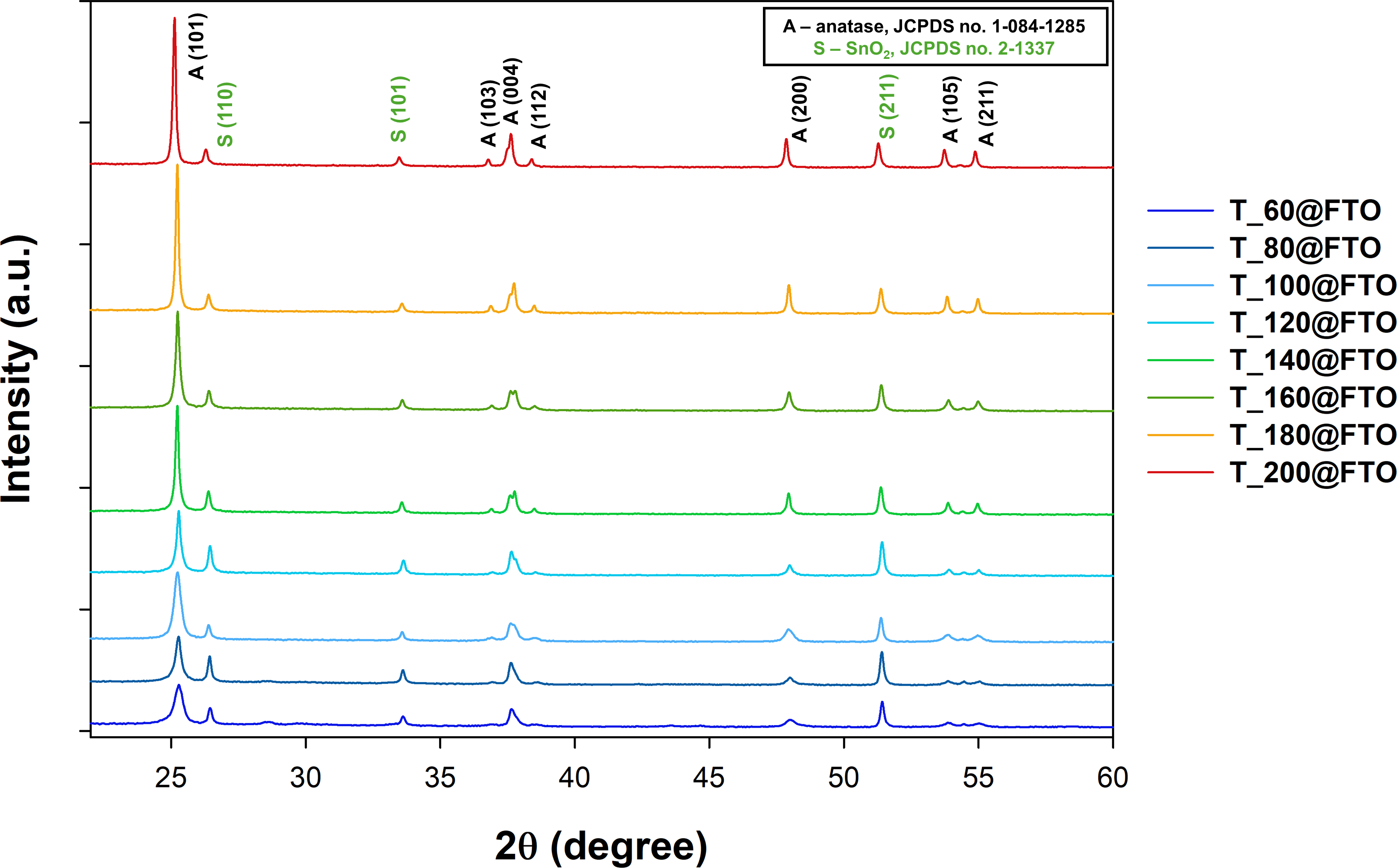


**Figure S6.** The XRD patterns registerd for the materials deposited at FTO substrates.


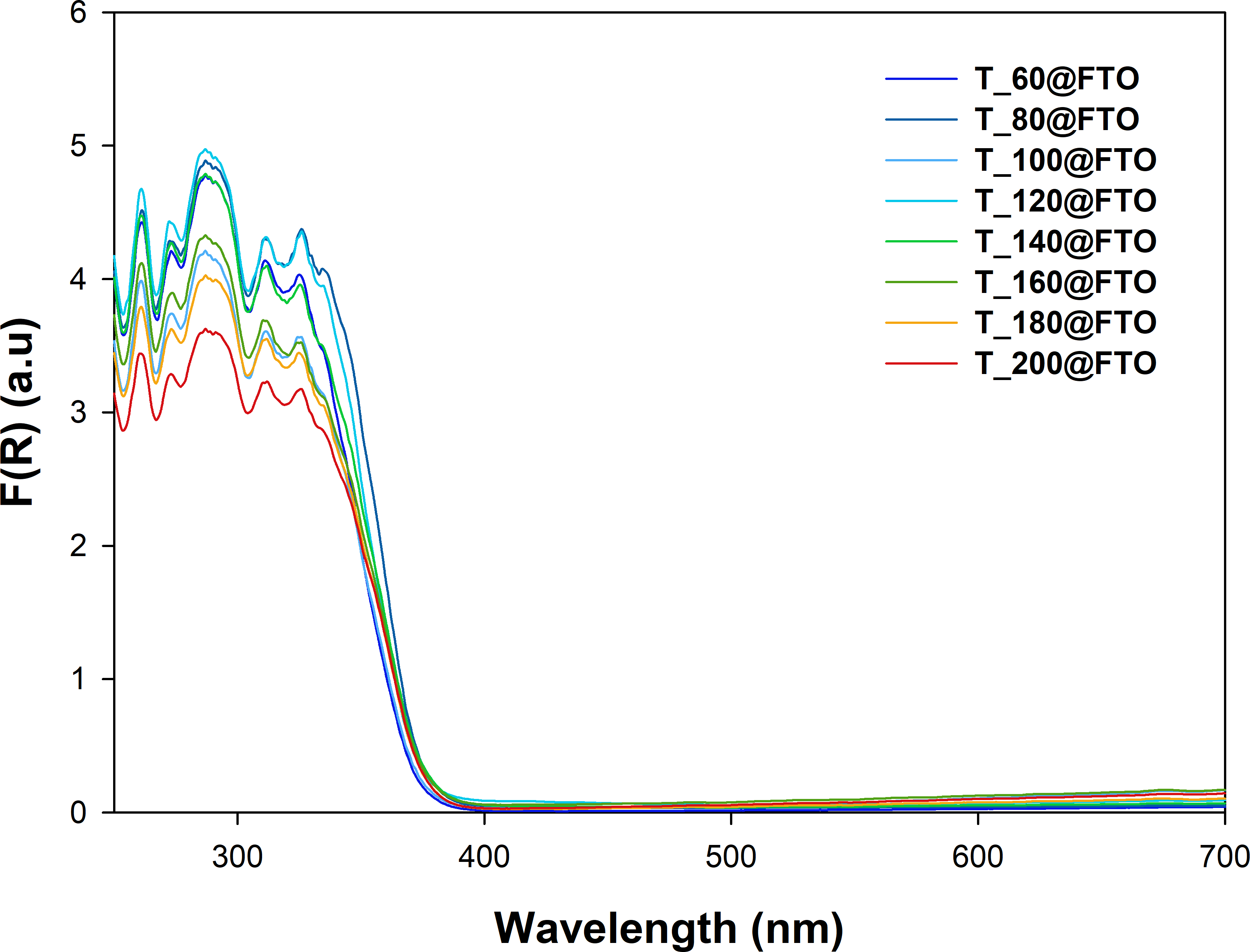


**Figure S7.** DRS spectra registered for the materials deposited at FTO substrates.


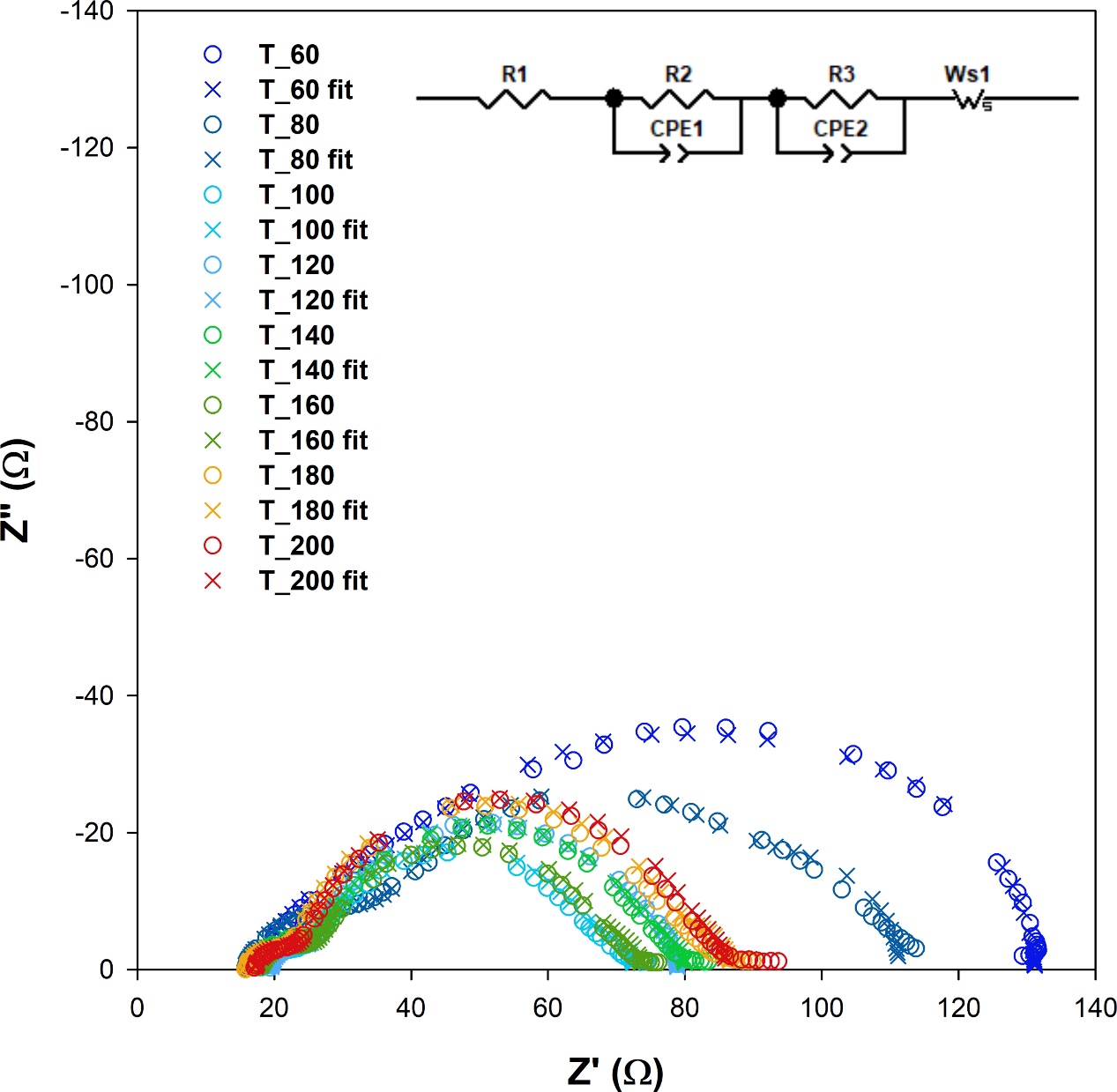


**Figure S8.** Nyquist plots of impedance spectroscopy results were registered for investigated DSSCs, and the equivalent circuit was used to fit the results (insert).


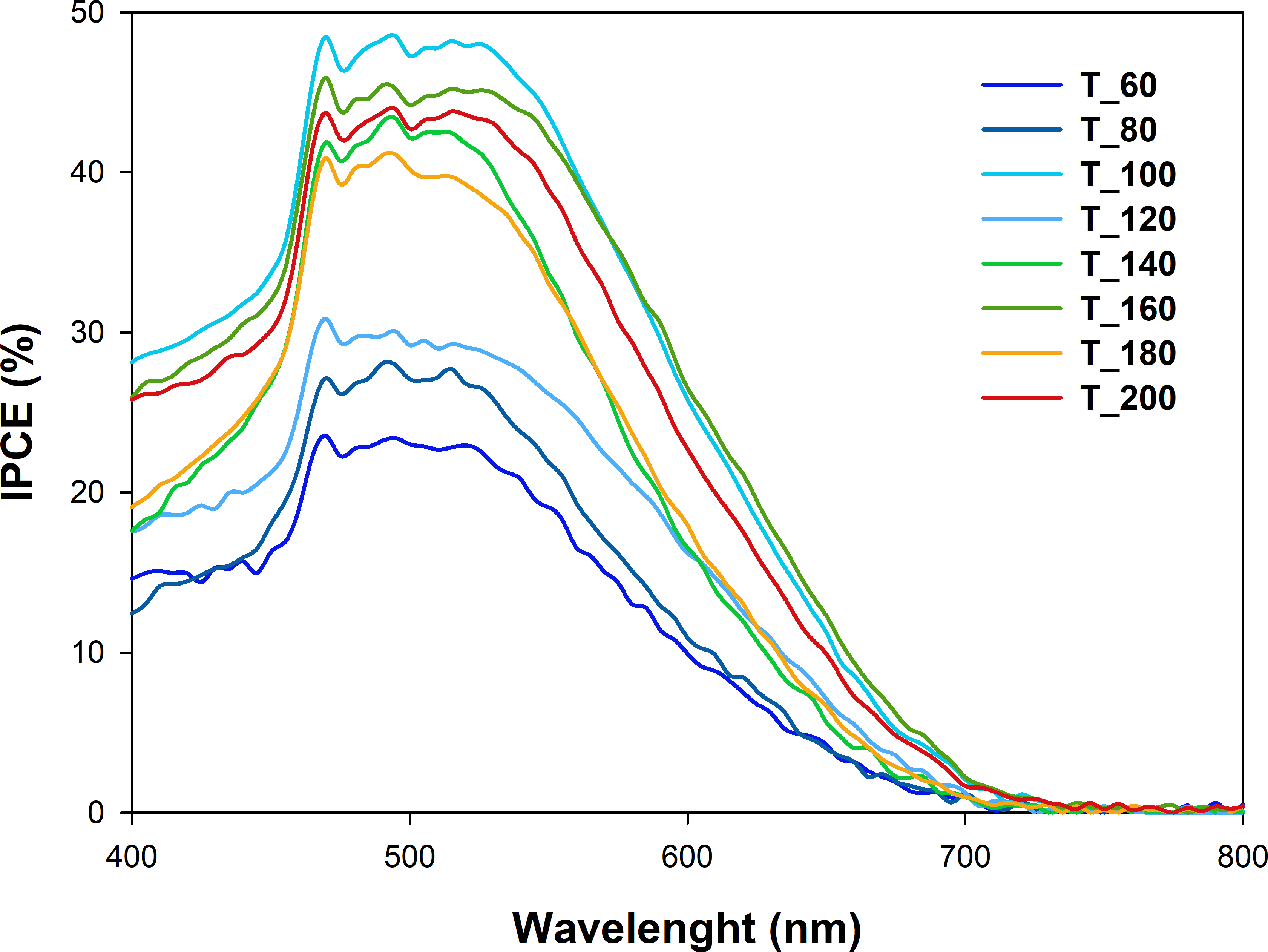


**Figure S9.** IPCE spectra registered for investigated DSSC devices.

**Table S4.** Impedance spectroscopy results

| Sample | R_1_ (Ω) | R_2_ (Ω) | R_3_ (Ω) | τ (ms) |
| --- | --- | --- | --- | --- |
| T_60 | 16.9 | 24.2 | 54.2 | 5.1 |
| T_80 | 15.6 | 20.5 | 51.3 | 7.8 |
| T_100 | 18.1 | 8.2 | 39.1 | 6.4 |
| T_120 | 19.5 | 5.9 | 47.7 | 4.2 |
| T_140 | 17.1 | 9.1 | 47.6 | 5.1 |
| T_160 | 17.9 | 8.6 | 41.4 | 6.4 |
| T_180 | 15.9 | 6.9 | 55.6 | 6.4 |
| T_200 | 17.1 | 6.7 | 57.1 | 6.4 |
